# L-type calcium channel blockers at therapeutic concentrations are not linked to CRAC channels and heart failure

**DOI:** 10.1080/10641963.2025.2515924

**Published:** 2025-07-12

**Authors:** Gary S. Bird, Yu-Ping Lin, Charles J. Tucker, Geoffrey Mueller, Min Shi, Sandosh Padmanabhan, Anant B. Parekh

**Affiliations:** a Molecular and Cellular Biology Laboratory, National Institute of Environmental Health Sciences, NIH, Research Triangle Park, NC, USA; b Department of Biotechnology and Bioindustry Sciences, National Cheng Kung University, Tainan City, Taiwan; c Fluorescence Microscopy and Imaging Center, National Institute of Environmental Health Sciences, NIH, Research Triangle Park, NC, USA; d Nuclear Magnetic Resonance Research Laboratory, National Institute of Environmental Health Sciences, NIH, Research Triangle Park, NC, USA; e Biostatistics & Computational Biology Branch, National Institute of Environmental Health Sciences, NIH, Research Triangle Park, NC, USA; f BHF Glasgow Cardiovascular Research Centre, School of Cardiovascular and Metabolic Health, University of Glasgow, Glasgow, UK

**Keywords:** Hypertension, amlodipine, Ca^2+^ channels, heart failure, Ca^2+^ release-activated Ca^2+^ (CRAC) channels, store-operated calcium entry (SOCE)

## Abstract

Amlodipine has been used as a front-line anti-hypertensive therapy for decades by virtue of blocking voltage-operated calcium channels with high affinity and specificity. Recently, the safety of amlodipine has been questioned, as it was reported to activate Ca^2+^ release-activated Ca^2+^ (CRAC) channels and increase the risk of heart failure. Here we show, using a variety of approaches, that amlodipine does not activate CRAC channels at therapeutic concentrations. Combined with our previous meta-analysis, our study should reassure physicians that amlodipine should continue to be prescribed for treating hypertension.

## Introduction

Globally, around one in five adults is diagnosed with the “silent killer” hypertension, which significantly increases the risk of myocardial infarction, heart failure, stroke, retinopathy and kidney disease (1). One hundred and sixteen million Americans are thought to have hypertension or are taking medication for its treatment (2). The European Commission reports 1 in 5 Europeans are hypertensive (3), the WHO estimates 46% of adults over 25 years of age in Africa have high blood pressure (4) and between 15% and 35% of urban adult populations of Asia are hypertensive (5). Several different classes of drug are used to treat hypertension, and L-type calcium channel blockers (LCCBs) are a universal first-line treatment (6). The most effective and widely prescribed LCCB is the dihydropyridine amlodipine. In India, where amlodipine is the most widely administered anti-hypertensive agent (7), the drug costs less than $3 for an entire month’s supply, making it affordable to low-income families. Challenging the safety of amlodipine and other LCCBs therefore impacts directly on the health and lifespan of almost 1 billion people, as well as their families. Any such challenge must have a rigorous scientific basis.

This well-established view has been recently challenged (8,9) based on the observations that i) amlodipine increases the risk of heart failure compared with other anti-hypertensive drugs, obtained from analysis of patients’ records and ii) amlodipine activates vascular remodeling by opening store-operated Ca^2+^ entry (SOCE), mediated through Ca^2+^ release-activated Ca^2+^ (CRAC) channels. Store-operated calcium entry (SOCE), is a crucial calcium influx pathway triggered by depletion of intracellular calcium stores in the endoplasmic reticulum (ER), leading to STIM protein aggregation and subsequent opening of Orai proteins, the pore forming subunits of CRAC channels.

LCCBs such as amlodipine were reported to activate CRAC channels in various non-excitable cells, including HEK293 cells, when applied at micromolar concentrations, a conclusion drawn mainly by using the calcium indicator dye Fura-2 (8,9). It was also reported that 0.5 µM amlodipine, which marginally increased Ca^2+^ signals (8), interacted synergistically with platelet-derived growth factor to increase proliferation of cultured vascular smooth muscle cells (8,9). The authors concluded: “Our data indicate caution against the use of LCCBs in elderly patients or patients with advanced hypertension and/or onset of cardiovascular remodelling, where levels of STIM and ORAI are elevated” (8).

This conclusion by Johnson et al. (8) calling into question the safety of amlodipine was surprising. Amlodipine is a frontline global treatment for hypertension (1,6). It is well-tolerated with few side effects, has reasonable oral availability and favorable pharmacokinetic properties that enable single dosing daily. In contrast to Johnson et al. (8) and Trebak et al. (9), multiple epidemiological studies have concluded that amlodipine is safe and effective (10–15). We conducted our own carefully controlled meta-analysis of published clinical trials and a prospective real-world analysis of patients prescribed single antihypertensive agents which both showed that dihydropyridines were not associated with increased heart failure or other cardiovascular disorders (16). Characterizing the actions of amlodipine at or above 0.5 µM and relating this to patients (8) is also a concern. Therapeutic unbound levels of amlodipine in the blood are typically in the sub- to low nM (0.7–36) range (17,18), with comatose concentrations being 0.245–0.490 µM (0.1–0.2 mg/L) and toxic levels at 0.215 µM^17^. These are all well below the lowest concentration of amlodipine that was reported to activate CRAC channels (8). Such low therapeutic levels are effective because amlodipine inhibits CaV1.2 Ca^2+^ channels in vascular smooth muscle with an IC_50_ in the low nM range (19), leading to vessel vasodilation and a decrease in blood pressure.

A further area of concern with the conclusions of Johnson et al. (8) comes from the use of the fluorescent dye Fura-2 to measure cytosolic Ca^2+^ in studies with amlodipine (16). Other groups have reported complex effects of amlodipine on Fura-2-based measurements. Asai et al. (20) found that amlodipine inhibited cytosolic Ca^2+^ signals measured with Fura-2 in a concentration-dependent manner, with near complete suppression at 10 µM (20). Quentin et al. (21) found substantial intracellular accumulation of amlodipine in organelles, particularly lysosomes, and this led to enhanced fluorescence emission following excitation at the same wavelengths used for Fura-2. Our recent study (16) is in good agreement with those earlier works that reported Fura-2 was unsuitable for measuring cytosolic Ca^2+^ in the presence of amlodipine. We found that amlodipine (above 0.5 µM) was autofluorescent in extracellular solution but became several fold-more fluorescent when it rapidly accumulated within intracellular compartments within seconds to minutes of exposure (16). Amlodipine washed out of cells very slowly, over tens of minutes. Therefore, the intracellular signal emanating from its compartmentalization cannot be removed during the time course of a typical cytosolic Ca^2+^ experiment. At the excitation wavelengths for Fura-2 (340 and 380 nm), the emission from intracellular amlodipine was considerably higher than for Ca^2+^-Fura-2, suppressing the latter signal. Using a longer excitation wavelength dye (Cal 520), we failed to see any Ca^2+^ influx to concentrations of amlodipine of 1 µM or less (16). At higher concentrations, we observed polypharmacological effects of amlodipine including Ca^2+^ release from thapsigargin-sensitive Ca^2+^ stores and inhibition of store-operated Ca^2+^ entry. These observations are also supported by a substantial body of literature demonstrating that microMolar concentrations of LCCBs block store-operated Ca^2+^ entry (22–27) rather than activate the pathway, as reported (8). LCCBs such as amlodipine have also been found to release Ca^2+^ from the endoplasmic reticulum, subsequently reducing Ca^2+^ release to thapsigargin, and to inhibit cell proliferation in vascular smooth muscle (27–32) instead of stimulating it (8).

In light of the number of people who take amlodipine and that the drug is the favored treatment in developing countries due to it being off patent, low cost and with few reported side effects, it is important to address the issue of amlodipine mechanism of action and whether it increases heart failure. We have carried out several new experiments which we believe bring clarity to the issues being discussed (33). Our new data demonstrate that Fura-2 cannot be used in combination with amlodipine due mainly to the high fluorescence of amlodipine within cells, which is not possible to subtract by removal of amlodipine autofluorescence in external solution. We demonstrate that the fluorescence from intracellular amlodipine occurs over the entire excitation spectrum of Fura-2. Using more appropriate dyes that are not contaminated by amlodipine fluorescence, namely Fluo-4, Cal 420 and Fura Red, we have consistently failed to detect Ca^2+^ influx at 0.5 µM or 20 µM amlodipine. These results cast serious doubts on the view that L-type Ca^2+^ channel blockers activate store-operated Ca^2+^ channels at therapeutic concentrations and increase the risk of heart failure.

## Materials and methods

### Cell culture

HEK293 cells (ATCC, CRL 1573) were cultured in Dulbecco’s minimum essential medium (DMEM) supplemented with 10% heat-inactivated fetal bovine serum and 2 mM glutamine and maintained in a humidified 95% air, 5% CO_2_ incubator at 37°C. In general, in preparation for cytosolic Ca^2+^ measurements, fluorescence imaging or confocal microscopy, cells were subcultured onto 30 mm round glass coverslips (#1.5 thickness) and maintained in culture for an additional 36–48 h. Experiments were performed with a HEPES-buffered salt solution (HBSS in mM: NaCl 120; KCl 5.4; MgCl_2_ 0.8; HEPES 20; CaCl_2_ 1.8 and glucose 10, with pH 7.4 adjusted by NaOH).

### Cell transfection

HEK293 cells were plated in a 6-well plate on Day 1. On Day 2, cells were transfected using Lipofectamine 2000 (Invitrogen; 2 ml per well) with cDNA (0.5 µg/well) for EYFP-tagged STIM1 and CFP-tagged Orai1. After a 6 hr incubation period, the medium bathing the cells was replaced with complete DMEM and maintained in culture. On Day 3, cDNA treated cells were transferred to 30 mm glass coverslips in preparation for single-cell Ca^2+^ measurements (as described below), which were performed on Day 4 or 5. At the start of each Ca^2+^ measurement, cells overexpressing EYFP-STIM1 and CFP-Orai1 were identified by the fluorescence from EYFP-STIM1.

### Isolation of human peripheral blood mononuclear cells (PBMCs)

Blood samples were collected from volunteer “control” and “test” subjects at the NIEHS Clinical Research Unit. Volunteers gave their full consent at three independent stages to the research team, the clinical research center and to the lead physician. Approval from the local ethics committee was therefore waived. Control samples were taken, with full consent, from members of the research team. To isolate PBMCs, whole blood samples were collected into a BD Vacutainer® CPT™ Mononuclear Cell Preparation Tube containing Sodium Heparin (BD Bioscience, Catalog No: 362753). The samples were centrifuged at 1800 × g for 5 min, resulting in mononuclear cells being located in between plasma and the polyester gel. The layer of mononuclear cells was transferred to a 50 ml falcon tube, and the cells washed twice with PBS. PMMCs were then resuspended in HBSS containing 2 mM CaCl_2_ (HBSS: 145 mM NaCl, 2.8 mM KCl, 2 mM MgCl_2_, 10 mM D-glucose, 10 mM HEPES, 0.1 mM EGTA, 2 mM CaCl_2_ pH 7.4 adjusted with NaOH) and quickly plated on 35 mm glass bottom dish (MATTEK, Part No: P35G–1.5–14-C). The MATTEK dish was then placed on the stage of a Zeiss AxioObserver epifluorescence microscope (Carl Zeiss Inc, Oberkochen, Germany) equipped with a Zeiss Plan-Apochromat objective (20×/0.8 NA) and a Colibri 7 LED light source. To detect and visualize amlodipine fluorescence, the 385 nm LED was used for excitation with a 370–400 nm excitation filter coupled with an emission filter of 500–530 nm. A Hamamatsu ORCA Flash C11440 camera was used to collect fluorescence images with binning set to 2 × 2 and a 50 ms exposure. Additionally, time-series images were acquired with Definite Focus engaged to eliminate focal drift. Images were taken every 1 min. The time between the onset of centrifugation and the start of the imaging was <8 min.

### Single cell calcium measurements with UV ratiometric calcium indicator

Fluorescence measurements were made with HEK293 cells loaded with the Ca^2+^-sensitive dye, Fura-5F, as described previously (16,34). Briefly, cells plated on 30 mm round coverslips and mounted in a Teflon chamber were incubated in DMEM with 1 μM acetoxymethyl ester of Fura-5F (Fura-5F/AM, Setareh Biotech, Eugene, OR) at 37°C in the dark for 25 min. Cells were bathed in HEPES-buffered salt solution (HBSS in mM: NaCl 120; KCl 5.4; Mg_2_SO_4_ 0.8; HEPES 20; CaCl_2_ 1.0 and glucose 10 mM, with pH 7.4 adjusted by NaOH) at room temperature. Nominally Ca^2+^-free solutions were HBSS with no added CaCl_2_. Fluorescence images of the cells were recorded and analyzed with a digital fluorescence imaging system (InCyt Im2, Intracellular Imaging Inc., Cincinnati, OH). Fura-5F fluorescence was monitored by alternatively exciting the dye at 340 nm and 380 nm and collecting the emission wavelength at 520 nm. Changes in cytosolic Ca^2+^ are expressed as the “Ratio (F340/F380)” of Fura-5F fluorescence due to excitation at 340 nm and 380 nm (F340/F380). Before starting the experiment, regions of interests identifying cells were created and 25 to 35 cells were monitored per experiment. For experiments with cells overexpressing EYFP-STIM1 and CFP-Orai1, regions of interests were created by imaging the cells and confirming EYFP-Stim1 fluorescence. Ca^2+^ measurements were performed in HBSS supplemented with 1 mM CaCl_2_.

### Visualizing amlodipine fluorescence and cell energy depletion

HEK293 and RBL-2H3 cells were plated on 35 mm glass bottom dish (MATTEK, Part No: P35G–1.5–14-C) and maintained in culture for 24 h so that, on the day of experiments, cell confluency was 70–80%. To “energy deplete” cells, cells were bathed in glucose-free HBSS containing a Rotenone/Antimycin cocktail (1 µM, Agilent Technologies) and 10 mM 2-deoxy-D-glucose for 60 min at room temperature. After this incubation period, the bathing solution was switched to HBSS and the MATTEK dish placed on the stage of a Zeiss AxioObserver epifluorescence microscope (Carl Zeiss Inc, Oberkochen, Germany) equipped with a Zeiss Plan-Apochromat objective (20×/0.8 NA) and a Colibri 7 LED light source. To detect and visualize amlodipine fluorescence, the 385 nm LED was used for excitation with a 370-400 nm excitation filter coupled with an emission filter of 500–530 nm. A Hamamatsu ORCA Flash C11440 camera was used to collect fluorescence images with binning set to 2 × 2 and a 50 ms exposure. Additionally, time-series images we acquired with Definite Focus engaged to eliminate focal drift. Images are taken every 2 min for 30 min.

### Cytosolic Ca^2+^ measurements using single wavelength indicators

Cytosolic Ca^2+^ was monitored in HEK293 cells using three different single wavelength calcium indicators, Fluo-4, Cal 520 and Fura Red.

Cytosolic Ca^2+^ was monitored in Fluo-4-loaded and Cal 520-loaded HEK293 cells using a fluorometric imaging plate reader (FLIPR^TETRA^; Molecular Devices, Inc., Sunnyvale, CA). HEK293 cells were plated 24 h before use on polyLysine-coated 96-well plates at 40 000 cells/well. On a single 96-well plate, cells were then loaded with either the visible wavelength indicator Fluo-4/AM (Rows A-D; 4 μM Fluo-4/AM) or Cal 520 (Rows E-H; 4 μM Cal 520/AM) by incubation in complete DMEM for 45 min at 37°C. After the indicator loading period, cells were washed twice in nominally Ca^2+^-free HBSS and then bathed in HBSS supplemented with 2 mM CaCl_2_. The 96-well plate was placed on the FLIPR observation stage, and the indicator loaded cells were excited at 488 nm with emission fluorescence detected by a cooled charge coupled device camera via 510–570-nm bandpass filter. Experiments were carried out at room temperature. Changes in cytosolic Ca^2+^ are expressed as the “F/Fo” of Fluo-4 or Cal 520 fluorescence, whereby the time course of fluorescence intensities (F) was divided by the initial fluorescence intensity recorded at the start of the experiment (Fo).

Cytosolic Ca^2+^ was monitored in Fura Red-loaded HEK293 cells on the stage of a Zeiss LSM780 confocal microscope (Carl Zeiss Inc, Oberkochen, Germany). In preparation for Ca^2+^ measurements, HEK293 cells were cultured on 30 mm round coverslips. On the day of the experiment, the coverslips were mounted in a Teflon chamber and the cells loaded with 2 µM Fura Red/AM by incubation in complete DMEM for 45 min at 37°C. After indicator loading, cells were washed twice in nominally Ca^2+^ -free HBSS and then bathed in HBSS supplemented with 2 mM CaCl_2_. The chamber was then mounted on the stage of the microscope equipped with a Zeiss Plan-APOCHROMAT 10× objective (N.A. 0.45). Fluorescence images were collected with a pinhole set at 7–9 Airy unit (equivalent to a z-slice of 50 µm). Regions of interest identifying cells were created and 40 to 50 cells were monitored per experiment. Two image channels were acquired in a time series with the Zeiss Zen Black software (version 2.3 SP1) to separately monitor changes in Fura Red fluorescence (excitation 488 nm, emission 601–706 nm) and the accumulation of Amlodipine Besylate (excitation 355 nm, emission 475–550 nm). Subsequently, the Fura Red data was expressed as “F/Fo,” the time course of the Ca^2+^ signal (F) was divided by the initial Ca^2+^ signal recorded at the start of the experiment (Fo). In contrast to Cal 520 and Fluo-4, an increase in cytosolic Ca^2+^ causes a decrease in Fura Red fluorescence. However, in common with Cal 520 and Fluo-4, the fluorescence associated with the accumulation of intracellular amlodipine (insert to panel C) does not interfere with Fura Red.

### D1ER and monitoring Ca^2+^_ER_

To directly measure changes in the levels of ER Ca^2+^ content (Ca^2+^_ER_) we used HEK293 cells stably expressing the ER-targeted D1ER cameleon (35). These cells were generated by transfecting HEK293 cells using Lipofectamine 2000 (Invitrogen; 2 µl per well) with D1ER cDNA (0.5 µg/well; a generous gift from Dr Amy E. Palmer). After a 6 hr incubation period, the medium bathing the cells was replaced with complete DMEM and maintained in culture. Subsequently, the population of HEK-D1ER expressing cells was routinely sorted and enriched by flow cytometry.

In preparation for Ca^2+^_ER_ measurements, HEK-D1ER cells were cultured plated on 30 mm round coverslips. On the day of the experiment, the coverslips were mounted in a Teflon chamber, the cells bathed in HBSS that was nominally Ca^2+^-free (no added CaCl_2_), and the chamber mounted on the stage of a Zeiss LSM 780 confocal microscope equipped with a 20× objective (N.A. 0.8W). Fluorescence images were collected with a pinhole set at 10 Airy unit (equivalent to a z-slice of 16 µm). Under these image capture settings, the number of cells in the field of view were 150–200. Three-channel images of D1ER were acquired in a time series with the Zeiss Zen Black software (version 2.3 SP1). The three channels were: Donor channel (excitation 458 nm, emission 464–500 nm), FRET channel (excitation 458 nm, emission 526–597 nm) and a Ratio image that was generated with the following calculation: ((FRET +1)/(Donor +1)) * 10. After experiments were completed, the three-channel image was then opened in FIJI (v1.54f) and a macro used to automate the analysis of the time-series images. Specifically, the macro would: (i) open the image, (ii) select the donor channel and run the smooth command twice, (iii) after the smooth command, a threshold of the donor channel was used to create a mask of the time series of areas above 25 counts, (iv) a “Boolean AND” command was used with this mask and the Ratio channel to create a new image where the background (non-cell area) now has a pixel value of 0, (v) finally, a threshold was set to this new image to measure the average intensity of pixels greater than 0 thus yielding the mean intensity of only the D1ER expressing cells in the Ratio image. Subsequently, data from the Ratio image was expressed as the “F/Fo” of D1ER signal, whereby the time course of the Ca^2+^_ER_ ratio signal (F) was divided by the initial Ca^2+^_ER_ ratio signal recorded at the start of the experiment (Fo).

### YFP-STIM1 puncta formation

To measure the effects of amlodipine and carbachol on STIM1 puncta formation, we used HEK293 cells stably expressing YFP-STIM1. These cells were generated by transfecting HEK293 cells using Lipofectamine 2000 (Invitrogen; 2 µl per well) with YFP-STIM1 (0.5 µg/well). After a 6 hr incubation period, the medium bathing the cells was replaced with complete DMEM and maintained in culture. Subsequently, the population of HEK-YFP-STIM1 expressing cells was routinely sorted and enriched by flow cytometry.

In preparation for monitoring puncta formation, HEK-YFP-STIM1 cells were cultured plated on 30 mm round coverslips. On the day of the experiment, the coverslips were mounted in a Teflon chamber, the cells bathed in HBSS supplemented with 2 mM CaCal_2_, and the chamber mounted on the stage of a Zeiss LSM 880 confocal microscope equipped with a 40× C-Apochromat objective (N.A. 1.2W). Fluorescence images were collected with a pinhole set at 1.66 Airy unit (equivalent to a z-slice of 1.4 µm). Images of YFP-STIM1 (excitation 488 nm, emission 490–553 nm) was acquired in a time series with the Zeiss Zen Black software (version 2.3 SP1).

### Measuring NMR characteristics of amlodipine besylate in the absence and presence of fetal bovine serum (FBS)

NMR samples were prepared by diluting FBS and Amlodipine Besylate into phosphate buffered saline (PBS) with 10% 2H_2_O, and 50 mM Sodium trimethylsilylpropanesulfonate (DSS). NMR data were acquired on a 600 MHz Agilent DD2 spectrometer equipped with a cryogenically cooled probe using the Agilent “tnnoesy” pulse sequence with 1 s acquisition time, 1 s recycle time and 100 ms mixing time. Peak heights of Amlodipine and Besylate were fit with VNMRSYS (Agilent) and Chenomx (Alberta, Canada) software programs to measure concentrations. The spectral characteristics of besylate were not affected by the presence of FBS and provided a convenient internal control to calibrate the change in concentration of amlodipine as the % content of FBS was increased. NMR peak intensities were therefore scaled to the besylate peak height.

### Measuring spectral characteristics of amlodipine besylate and the fluorescent calcium indicator Fura-2

HEK293 cells were plated 48 h before use on polyLysine-coated 12-well plates. Cells were cultured in only eight wells, leaving the remaining four wells available for monitoring the spectral properties of bathing solution. Of the eight wells containing cells, only four wells of cells were incubated in DMEM with 4 μM Fura-2/AM (Fura-2/AM, Molecular Probes, USA) at 37°C in the dark for 45 min. All other wells just contained DMEM. In preparation for the spectral scan, the cells were bathed at room temperature in HEPES-buffered salt solution (HBSS in mM: NaCl 120; KCl 5.4; Mg_2_SO_4_ 0.8; HEPES 20; CaCl_2_ 10 and glucose 10, with pH 7.4 adjusted by NaOH) and then treated with one of the three different conditions: (1) 0.1% DMSO, (2) 2 µM TG, (3) 20 µM amlodipine. After a 15-min incubation period, fluorescence excitation spectra scan (Excitation 330–400 nm, Emission 510 nm) was recorded and analyzed using a VANTAstar fluorometer (BMG Labtech, Cary, NC, USA). For each of the three conditions described above, spectral data was recorded from wells with “Fura-2-loaded cells,” “cells only, no Fura-2” and “solutions.”

### Materials

Thapsigargin was purchased from Alexis (San Diego, CA), Fura-5F/AM Setareh Biotech (Eugene, OR), Fluo-4/AM and Fura-2/AM (Invitrogen, USA), Cal 520/AM (ATT Bioquest, CA), heat inactivated Fetal Bovine Serum (Gibco FBS; Thermo Fisher, USA). Amlodipine Besylate and Diltiazem from Selleck Chemicals (Houston, TX), 30 mm round #1.5 coverslips from Bioptechs (Butler, PA), BioCoat Poly-D-Lysine 96-well Black/Clear Flat Bottom TC-treated Microplates (Corning, USA).

*Statistics*.

Data are presented as mean ± SEM, and statistical evaluation on raw data was carried out using ANOVA and Tukey’s multiple comparisons test (GraphPad Prism).

## Results

### Quantitative analyses of cytosolic Ca^2+^ measurements with Fura-5F when amlodipine is present

It has been proposed (8,9) that the effect of amlodipine and other LCCBs on vascular remodeling occurs through the activation of store-operated Ca^2+^ channels via an action on the ER, leading to STIM protein aggregation (independent of store depletion) and subsequent opening of CRAC channels. Gating of CRAC channels was shown at 20 µM amlodipine, although tiny Ca^2+^ influx was reported at the lowest concentration tested, 0.5 µM (8). Most of the Ca^2+^ measurements used to draw this conclusion were based on the use of the fluorescent indicator dye Fura-2 (8).

Several reports have measured cytosolic Ca^2+^ with Fura-2 and assessed the impact of amlodipine on the Ca^2+^ signals. However, previous studies raised concerns with the use of Fura-2 to measure cytosolic Ca^2+^ in the presence of amlodipine (19). A major artifact is that amlodipine accumulates inside organelles such as lysosomes within minutes, and this results in a severalfold increase in drug fluorescence emission at similar excitation wavelengths to those used for Fura-2 (20). Our recent data confirmed amlodipine is auto-fluorescent in solution, is excited at 340 and 380 nm wavelengths just like Fura-2, accumulates rapidly inside cells which increases its fluorescence several fold further, washes out slowly and yields emission signals that are several fold larger than those induced by Ca^2+^-Fura-2 (16,20). We therefore have designed a series of experiments to see whether Fura-2 can be combined with amlodipine and, if so, what precautions need to be taken.

**Figure 1.**
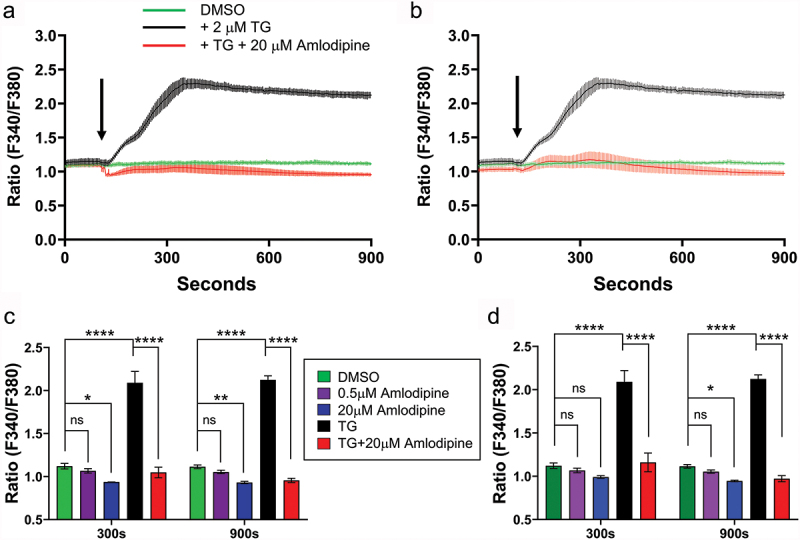
Amlodipine suppresses Ca^2+^ signals to thapsigargin in Fura-5F loaded cells. (a), Application of 20 µM amlodipine with 2 µM thapsigargin (red trace) resulted in suppression of the thapsigargin-evoked Ca^2+^ response (black trace). (b), Autofluorescence signals measured in extracellular solution at 340 nm and 380 nm (± Amlodipine) were subtracted from the relevant traces in panel a and the new corrected “Ratio” plotted. Despite this “correction,” which Trebak et al. (9) describe as the method they used to eliminate amlodipine fluorescence, the thapsigargin-evoked Ca^2+^ signal remained suppressed. This is because the correction fails to address the major problem of intracellular amlodipine fluorescence. All data are mean ± SEM for *n* = 3 experiments. (c,d), Aggregate data for time points 300 sec and 900 sec in panels a and b are summarized and data were compared by ANOVA (ns denotes not significant, *p≤ .05, **p ≤ .01, ****p ≤ .0001). Note also that amlodipine was co-applied with thapsigargin yet still suppressed Ca^2+^ signals to the latter. This shows that amlodipine accumulates intracellularly sufficiently rapidly to supress Ca^2+^ signals induced at the same time as amlodipine exposure. STIM1 and Orai1 were overexpressed to increase the size of store-operated Ca^2+^ entry.

The impact of amlodipine on cytosolic Ca^2+^ signals is shown in Figure 1. To enhance the ability to detect small changes in cytosolic Ca^2+^ signals, we overexpressed STIM1 and Orai1, the two key components of the CRAC channel. We measured Ca^2+^ signals with Fura-5F, which is identical to Fura-2, but has lower affinity for Ca^2+^ and avoids buffering and masking small changes in Ca^2+^ signals. Cells were stimulated with a maximally effective concentration of the SERCA pump blocker thapsigargin in the continuous presence of external Ca^2+^. Inhibition of SERCA pumps leads to unopposed leak of Ca^2+^ from the ER, raising cytosolic Ca^2+^ transiently. Following store depletion, store-operated CRAC channels open, leading to a large and sustained rise in cytosolic Ca^2+^ as Ca^2+^ enters the cell. In control cells, a prolonged rise in cytosolic Ca^2+^ was observed (black trace; Figure 1a). In contrast, in cells exposed to amlodipine together with thapsigargin, the cytosolic Ca^2+^ signal was almost completely suppressed (red trace; Figure 1a). Aggregate data, measured at 300 s and 900 s, are shown in Figure 1c.

Johnson et al. (8,9) attempted to correct the amlodipine fluorescence by subtracting the amlodipine autofluorescence from the extracellular solution. We therefore used this same method. The new corrected “Ratio” traces are plotted in Figure 1b. Despite the corrections, the thapsigargin-evoked Ca^2+^ signal remained suppressed (Figure 1b,d). This is because the correction does not address the dominant problem of intracellular amlodipine fluorescence, arising from accumulation within organelles (16,21). This signal is several fold larger than the amlodipine autofluorescence seen in extracellular solution.

**Figure 2.**
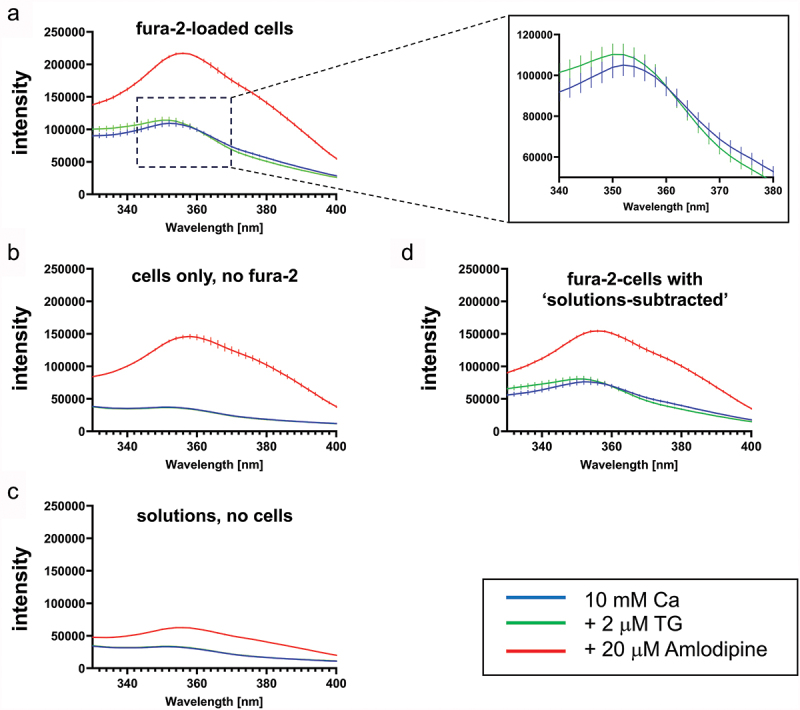
Fluorescence from intracellular amlodipine overwhelms the Fura-2 signal at all excitation wavelengths. (a), Compared to the “10 mM Ca” condition (blue trace), treating Fura-2-loaded HEK293 cells with thapsigargin increased the fura-2 emission at 340 nm excitation and reduced emission at 380 nm excitation. In the presence of 20 µM amlodipine (red trace), the excitation spectrum measured increased substantially and was enhanced over the entire excitation spectrum occupied by Fura-2. (b), As in panel a but cells had not been loaded with Fura-2. The spectrum to 20 µM amlodipine was only marginally smaller than that in panel a, demonstrating that intracellular accumulation of amlodipine was the dominant source of fluorescence. (c), Spectra are compared from extracellular solution alone. (d), Subtraction of panel c spectral data from the respective spectra in panel a should eliminate the amlodipine signal, according to the correction protocol applied by Johnson et al. (8), as discussed in Trebak et al. (9). Such correction is wholly inadequate because the amlodipine signal remains and is considerably larger than the Fura-2 responses to thapsigargin challenge. All data are mean ± SEM for *n* = 3 experiments. Excitation spectra (Excitation 330–400 nm, Emission 510 nm) were recorded under three conditions (indicated in the Figure) and from wells that contained: Panel a: “Fura 2-loaded cells,” Panel b: “cells only, no fura-2” and Panel c: “solutions”.

To see whether there was a wavelength that excited Fura-based dyes and not amlodipine, we obtained excitation spectra for Fura-2 and amlodipine, and these are shown in Figure 2. Fura-2 was used in order to reproduce the experiments by Johnson et al. (8). For Fura-2-loaded cells (panel A), the blue trace shows the Fura-2 spectrum in 10 mM external Ca^2+^, a concentration that was reported to correct for the amlodipine suppression of the Fura-2 signal (8). In the presence of thapsigargin (green trace), the 340 signal increases and the 380 decreases, with the Ca^2+^-insensitive isobestic point being ~360 nm, as expected for the Ca^2+^-independent wavelength and thus validating the experimental set-up. The red trace shows the effect of amlodipine in 10 mM Ca^2+^. The signal increases substantially at all wavelengths, including at 340 and 380 nm and, concerningly, also at the isobestic point. The amlodipine excitation spectrum overwhelms the Fura-2 fluorescence over the entire excitation range. Figure 2b shows amlodipine fluorescence in cells not loaded with Fura-2, and Figure 2c shows autofluorescence of amlodipine in external solution without cells. Autofluorescence from amlodipine in solution is much smaller than the fluorescence seen in cells, due to the intracellular accumulation of amlodipine, particularly in lysosomes that we and others have described (16,20). Figure 2d shows fluorescence spectra in Fura-2-loaded cells, after subtraction of the amlodipine autofluorescence in solution (Figure 2c). Subtracting the intrinsic amlodipine autofluorescence (8,9) should have generated a spectrum that falls within the bounds of the blue and green traces. This is clearly not the case (Figure 2d); the subtraction failed to remove the more significant signal arising from intracellular accumulation of amlodipine. We therefore conclude that amlodipine cannot be used in combination with Fura-2 to measure cytosolic Ca^2+^ signals.

### Measurement of cytosolic Ca^2+^ with other Ca^2+^-sensitive fluorescent dyes

**Figure 3.**
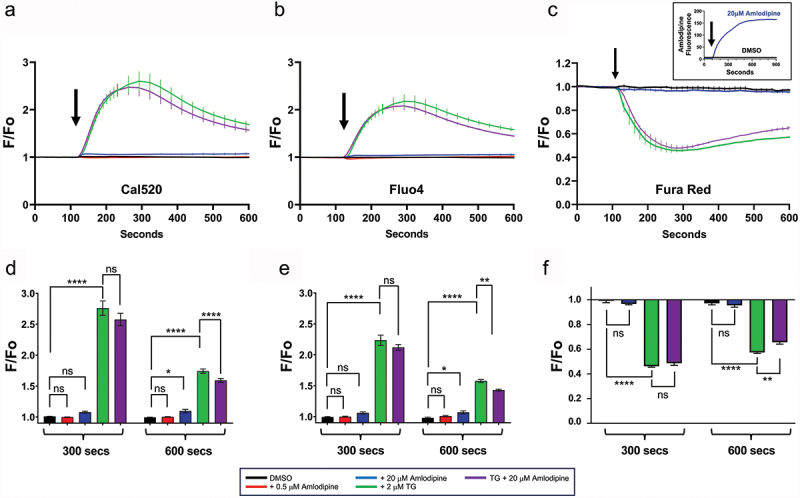
Amlodipine does not raise cytosolic Ca^2+^ when appropriate Ca^2+^ indicator dyes are used. (a), In Cal 520-loaded cells, 0.5 µM amlodipine failed to increase cytosolic Ca^2+^. 20 µM amlodipine evoked a miniscule response which was not significantly different from the DMSO control. Whilst thapsigargin elicited a substantial Ca^2+^ signal, it was reduced in the presence (co-addition) of 20 µM amlodipine at later time points. 20 µM amlodipine inhibits store-operated Ca^2+^ entry modestly, accounting for the reduced Ca^2+^ signal at later times, when the channels have opened. (b) and (c) Represent experiments as described in panel a, but Fluo-4 and Fura Red were used instead. The inset in c shows the intracellular accumulation of amlodipine, but which had no effect on the Fura Red signal. In all experiments, the extracellular CaCl_2_ concentration is 2 mM. Data are mean ± SEM for *n* = 6 experiments (panels a and b) and *n* = 3 experiments for panel c. (d–f), Aggregate data for time points 300 sec and 600 sec in panels a-c are summarized and data were compared by ANOVA (ns denotes not significant, *p ≤ .05, **p ≤ .01, ****p ≤ .0001).

**Figure 4.**
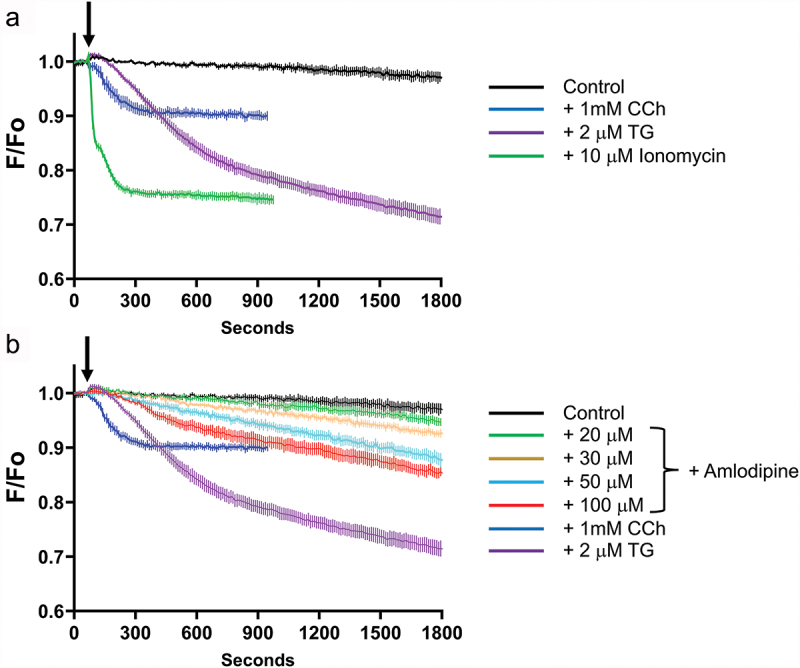
Amlodipine releases Ca^2+^ from the endoplasmic reticulum. (a), Validation of the measurements; Ionomycin (green trace), thapsigargin (purple trace) and carbachol (blue trace) all decrease Ca^2+^_ER_, which was measured directly by using the ER-targeted Ca^2+^-sensitive probe D1ER cameleon (b), amlodipine dose-dependently decreases Ca^2+^_ER_ at concentrations > 20 µM. All data are mean ± SEM for *n* = 6 experiments. The response to 20 µM amlodipine was significantly different from vehicle at times beyond 900 seconds (*p* < .05).

As Fura-2 is not a suitable dye for measuring cytosolic Ca^2+^ when combined with amlodipine, we sought other dyes that did not overlap with the excitation spectrum of the drug. Using the longer excitation wavelength dye Cal 520, we failed to detect Ca^2+^ influx to 0.5 µM (Figure 3a,d). A higher concentration of amlodipine (20 µM) evoked a very small Ca^2+^ rise, which was significantly different from vehicle only hundreds of seconds after application, and which likely involves weak store depletion (16) (see below (Figure 4)). In addition, 20 µM amlodipine partially inhibited store-operated Ca^2+^ entry to thapsigargin stimulation, as we and others have reported (16,22–27). 0.5 µM amlodipine caused a barely detectable Ca^2+^ rise (8). We found that 0.5 µM amlodipine failed to evoke a resolvable Ca^2+^ signal when Fluo-4 was used (Figure 3b,e). At 20 µM amlodipine, a small Ca^2+^ rise was detected, but only after ~600 s of exposure (Figure 3b,e). However, 20 µM amlodipine inhibited store-operated Ca^2+^ channels, as seen in the reduction on the amplitude of the Ca^2+^ plateau to thapsigargin (Figure 3b).

We also repeated these experiments with Fura Red, and measured changes in Fura Red fluorescence concomitant with intracellular accumulation of amlodipine. Amlodipine (20 µM) failed to evoke a Ca^2+^ signal (Figure 3c,f), although significant cytoplasmic buildup of amlodipine was apparent (inset in Figure 3c).

Collectively, using three different dyes (Cal 520, Fluo-4 and Fura Red), all of which are unaffected by overlap with amlodipine fluorescence, we have failed to detect Ca^2+^ entry through CRAC channels at 0.5 µM amlodipine. Higher concentrations have multiple effects, including Ca^2+^ release from intracellular stores as well as inhibition of CRAC channels.

### Amlodipine releases Ca^2+^ from the ER

Previously, we measured the extent of store release attributed to amlodipine by assessing the size of a subsequent response to the ER Ca^2+^-mobilizing agent ionomycin (16). Whilst 30 and 40 µM amlodipine caused clear store Ca^2+^ release, 20 µM amlodipine did not evoke a significant change (16). One possibility is that there is a threshold >20 µM amlodipine which is required for Ca^2+^ release. Alternatively, 20 µM amlodipine released Ca^2+^ from the ER but this was modest, so Ca^2+^ extrusion by the Gd^3+^-sensitive PMCA pump prevented cytosolic Ca^2+^ from rising. When the PMCA pump was blocked, significant Ca^2+^ release to 20 µM amlodipine treatment was revealed when Ca^2+^ extrusion was reduced (16).

Johnson et al. (8) measured ER Ca^2^ directly using GCaMP6–150, whereas we assessed ER Ca^2+^ content indirectly (16). We therefore repeated the experiments with a widely used ER Ca^2^ sensor (D1ER) that had been stably expressed in HEK293 cells. We first carried out a series of controls to validate the use of D1ER. Data are shown in Figure 4. Ionomycin and thapsigargin both evoked substantial ER Ca^2+^ release (Figure 4a). About 100 µM amlodipine also evoked prominent Ca^2+^ release (Figure 4b). Although this developed slowly, it was larger than the response to a maximally effective concentration of carbachol, which activates endogenous muscarinic receptors. 30 µM amlodipine evoked significant Ca^2+^ release and 20 µM amlodipine elicited slow and modest Ca^2+^ release that was nevertheless detectable and significantly different from vehicle (Figure 4b). Ca^2+^ release to 20 µM amlodipine is consistent with our earlier work and could explain the small amount of Ca^2+^ entry observed to this concentration of amlodipine at later time points (600 s) in Figure 3.

There are several reasons why our direct ER Ca^2+^ measurements differ from Johnson et al. (8). First, they transiently transfected GCaMP6–150, which would lead to variability in Ca^2+^ detector expression between cells, reducing the bandwidth of the recordings in cells with relatively low levels of ER-targeted probe. This would have been overlooked because their data are presented as F/F0 (8). Second, there is substantial variability in their experiments, for each condition (8), which could have masked a small decrease in ER Ca^2+^ stores to 20 µM amlodipine.

**Figure 5.**
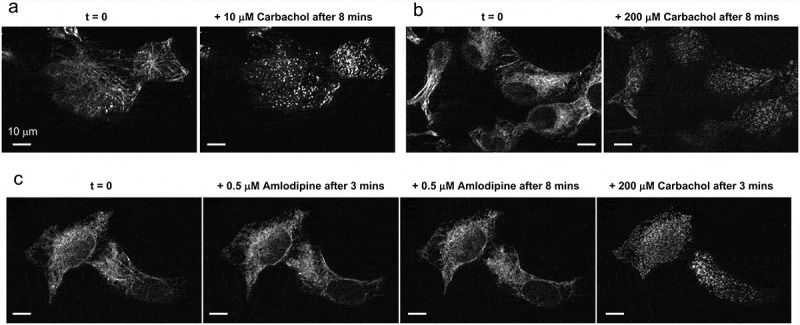
Carbachol, but not a low dose Amlodipine, induces YFP-Stim1 puncta formation. Treating HEK293 cells expressing YFP-STIM1 with 10 µM (a) or 200 µM carbachol (b) induces puncta formation. Treating cells with 0.5 µM amlodipine did not induce any puncta formation over a 8 min period of incubation (c), but subsequent exposure to 200 µM carbachol rapidly induced puncta formation. Data shown are representative images from 5 independent experiments. Scale bar shown is 10 µm.

STIM1 puncta formation is a crucial step in the activation of store-operated Ca^2+^ entry (SOCE). Activating phospholipase C-coupled receptors results in depletion of ER Ca^2+^ stores. STIM1, resident in the ER membrane, senses the drop in ER Ca^2+^ levels and undergoes a series of changes including oligomerization and the formation of puncta in peripheral ER just below the plasma membrane (PM). These puncta are clusters of STIM1 proteins that then interact with Orai1 channels in the plasma membrane, facilitating Ca^2+^ influx into the cell. Using HEK293 cells stably expressing STIM1 tagged with YFP (YFP-STIM1), we found that activation of the PLC-coupled muscarinic receptor with carbachol (10 µM or 200 µM, Figure 5a,b respectively) readily induced the formation of puncta. In contrast, a low dose of amlodipine (0.5 µM), a concentration that far exceeds levels measured in patients, failed to induce any puncta formation (Figure 5c).

### Estimate of amlodipine concentration in patients

The total concentration of amlodipine in the plasma of patients is in the range of 10–50 nM. With ~98% protein bound, the *free concentration* of amlodipine is 0.1–0.5 nM and it is the free concentration that determines the volume of distribution of a drug. Activation of store-operated Ca^2+^ entry was reported at µM concentrations of LCCBs, with marked Ca^2+^ entry seen at 20 µM (8), almost 4 orders of magnitude higher than therapeutic levels. However, it was suggested that LCCBs might accumulate inside the ER to reach levels of several µM (9), required for the activation of CRAC channels. We have directly tested this idea by taking advantage of the large, dose-dependent, amlodipine fluorescence that arises when it accumulates inside cells (16).

**Figure 6.**
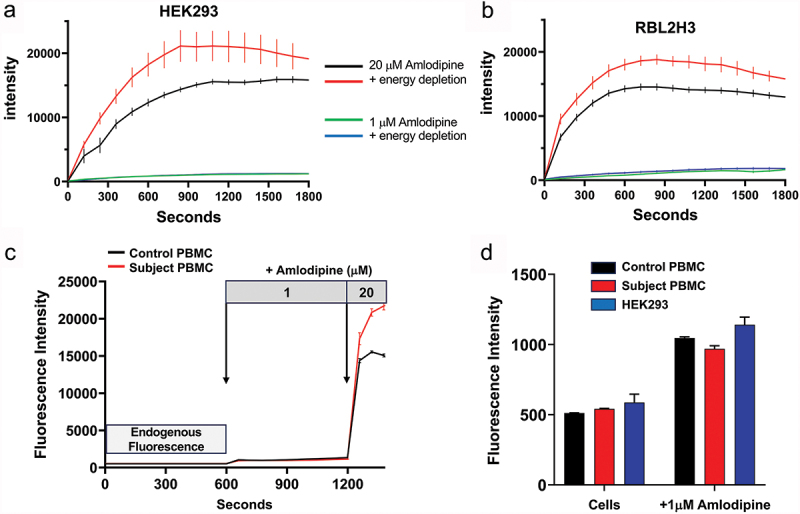
Amlodipine does not accumulate inside cells of hypertensive patients. (a), Intracellular accumulation of amlodipine was measured over time under energy replete conditions or after energy depletion (see methods) in HEK293 cells. Amlodipine was applied at either 1 µM (just above the threshold for detecting intracellular accumulation) or at 20 µM. (b), As in panel a but RBL-2H3 cells were used instead. In both cell types, loading was slightly faster and a little more extensive in energy-depleted cells, suggesting active transport might be required for removal of amlodipine. All data are mean ± SEM for *n* = 5 experiments. (c), Fluorescence of human peripheral blood mononuclear cells (PBMCs), taken from chronic hypertensive and control subjects, was measured (endogenous fluorescence). Then 1 µM or 20 µM amlodipine was sequentially applied to confirm whether the drug could indeed accumulate into primary human cells. In chronic hypertensive patients, who had been taking 5 mg amlodipine tablets daily for >15 years, no endogenous fluorescence was detectable above background autofluorescence (see panel D), but clear increases in fluorescence were observed when 1 µM and 20 µM amlodipine was subsequently applied. (d), Aggregate data from several experiments are compared. Cells denotes “endogenous fluorescence” (autofluorescence). +1 µM amlodipine shows the steady state fluorescence levels measured at 900 secs after application of exogenous amlodipine. Endogenous fluorescence in HEK293 cells were measured in parallel experiments under the same experimental condition. HEK293 were exposed to 1 µM amlodipine for 10 minutes and a similar increase in intracellular fluorescence occurred as was seen in subject PBMCs. Note that the HEK293 cells had not been exposed to amlodipine prior to application of 1 µM, unlike the subject PBMCs. Nevertheless, both cell types exhibited similar “endogenous fluorescence,” and accumulated amlodipine to similar extents. All data are mean ± SEM for *n* = 5 experiments.

With free amlodipine concentration in blood in the low nM range, intracellular accumulation to 10–20 µM could require active transport. Metabolic poisoning of cells failed to affect the rate and extent of intracellular accumulation of amlodipine (20 µM) in two different cell types (Figure 6a,b), ruling out active transport. In fact, accumulation was slightly higher after metabolic poisoning, suggesting that active transport might remove amlodipine from intracellular compartments.

Elderly patients or those with chronic hypertension often take amlodipine for many years. It is therefore possible that long-term exposure to amlodipine results in substantial intracellular accumulation. To test this directly, we drew blood from individuals who had been taking amlodipine continuously for >15 years for chronic hypertension. We isolated PBMCs and took precautions to minimize cells being exposed to amlodipine-free solution. After centrifugation for 5 min, cells were immediately placed under the objective of an epifluorescence microscope and imaged within 3 min. The half-time for washout of amlodipine is >45 min (16), and so the PBMCs would have maintained almost all intracellular amlodipine. We failed to see any intracellular fluorescence of amlodipine in the cells (Figure 6c), ruling out a possible intracellular accumulation of several log orders above plasma levels. However, when we applied 1 or 20 µM amlodipine to the PBMCs from patients or from healthy controls (Figure 6c,d), intracellular accumulation of amlodipine was immediately apparent. As a control, we used HEK293 cells that had no prior history of amlodipine exposure. HEK293 cells had a similar background fluorescence to cells from control or amlodipine-treated patients (Figure 6d), providing further evidence that amlodipine did not accumulate within the cells in the amlodipine-treated patients to any detectable extent.

### NMR measurements of free amlodipine levels in culture medium

**Figure 7.**
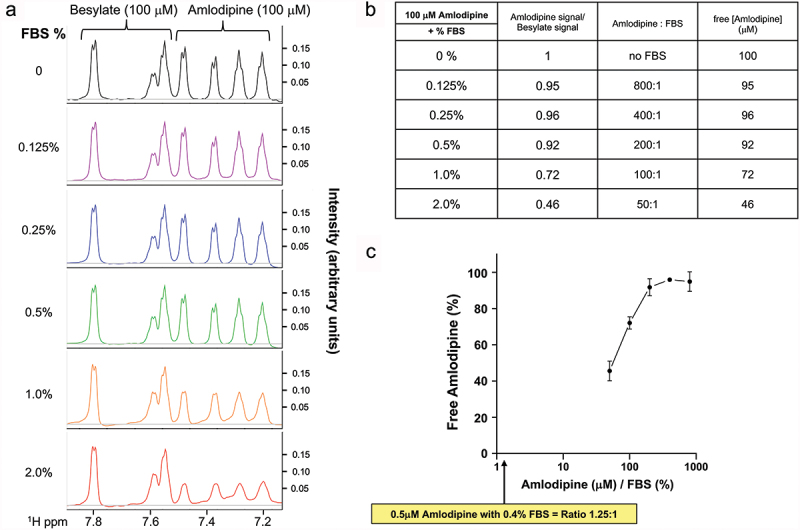
Amlodipine binds to fetal bovine serum. (a), Peak analysis of amlodipine (100 µM) and besylate (100 µM) in the presence of different % FBS. The spectral characteristics of besylate were not affected by the presence of FBS and provided a convenient internal control to calibrate the change in concentration of amlodipine, as the % content of FBS was increased. (b), Table shows how the amlodipine:besylate signal (derived from the peak analysis) changes as a function of % FBS. The corresponding free amlodipine concentration is shown in the right-hand column. The input amlodipine concentration was 100 µM throughout. These data were derived from three independent experiments. (c), the graph plots free amlodipine (% of total amlodipine) versus amlodipine:FBS ratio. Based on this plot, estimating the free amlodipine concentration under conditions of 0.5 µM amlodipine mixed with 0.4% FBS (an amlodipine:FBS ratio of 1.25:1) would suggest a free amlodipine of about ~ 1%, or 5 nM.

Previous studies have reported effects of amlodipine on cell growth and proliferation under cell culture conditions. The combination of 0.5 µM amlodipine, a concentration just above the threshold for activating CRAC channels, and 0.5 ng/ml PDGF was found to interact synergistically to increase myocyte proliferation and migration (8). These experiments were conducted in cell culture, in the presence of 0.4% fetal bovine serum (FBS) (8). Because amlodipine binding to plasma proteins is thought to be ~98% (36), we hypothesized that it should also bind to proteins in FBS, lowering the free, active concentration. We therefore assessed the extent of amlodipine binding to FBS by using NMR spectroscopy. 0.5 µM amlodipine was too low a concentration to provide reliable spectra. Nevertheless, because the amlodipine:FBS interaction follows the principles of mass action, what matters are the relative proportions of each agent. Prominent spectra were seen with 100 µM amlodipine besylate, with clear and well-separated peaks for besylate and amlodipine (Figure 7a). Increasing the % of FBS had no effect on the besylate signal but substantially reduced the amlodipine spectra (Figure 7a). We quantified the extent of amlodipine binding by integrating the area under the amlodipine spectra and comparing it with the corresponding and constant besylate signal. The Table in Figure 7b shows how this ratio changed with alterations in the amlodipine:FBS ratio, and then how this impacted the free amlodipine concentration. As the amlodipine:FBS ratio decreased, free amlodipine concentration also declined (Figure 7b). A plot of free amlodipine concentration against amlodipine:FBS ratio (Figure 7c) revealed an estimated free amlodipine concentration of ~1% of total amlodipine with a ratio of 1.25:1 (equivalent to the 0.5 µM amlodipine:0.4% FBS in ref 8). Therefore, the free amlodipine concentration under these experimental conditions is not 0.5 µM but ~5 nM. However, there may be some non-linearity in the extrapolations, and so the concentration indicated could be an overestimate. Nevertheless, even if one attributes an extremely improbable situation wherein no further amlodipine binds to FBS beyond a ratio of 50:1, the free amlodipine concentration would still only be 185 nM, a concentration well below the lowest concentration of amlodipine that was reported to activate store-operated Ca^2+^ entry. These results reveal that amlodipine binding to FBS needs to be taken into account when an LCCB is applied to culture medium.

## Discussion

Amlodipine is a frontline global treatment for hypertension (1,6) that is well-tolerated with few side effects, has reasonable oral availability and favorable pharmacokinetic properties that enable single dosing daily. Multiple epidemiological studies have concluded that amlodipine is safe and effective (10–15). Therapeutic unbound levels of amlodipine in the blood are typically in the sub- to low nM (0.7–36) range (17,18), and such low therapeutic levels are effective because amlodipine inhibits CaV1.2 Ca^2+^ channels in vascular smooth muscle with an IC_50_ in the low nM (19) range, leading to vessel vasodilation and a decrease in blood pressure.

This well-established view has been challenged (8,9) based on the observations that i) amlodipine increases the risk of heart failure compared with other anti-hypertensive drugs, obtained from analysis of patients’ records and ii) amlodipine activates vascular remodeling by opening store-operated Ca^2+^ channels through an action on the ER, leading to STIM protein aggregation and subsequent opening of Orai channels. Gating of CRAC channels was shown at 20 µM amlodipine in various cell types, including HEK293 cells, although tiny Ca^2+^ influx was reported at the lowest concentration tested, 0.5 µM. Most of the Ca^2+^ measurements used to draw this conclusion were based on the use of the fluorescent indicator dye Fura-2.

Our new data demonstrate that Fura-2 cannot be used in combination with amlodipine due mainly to the high fluorescence of amlodipine within cells, which cannot be subtracted by removal of amlodipine autofluorescence in external solution. We demonstrate that the fluorescence from intracellular amlodipine occurs over the entire excitation spectrum of Fura-2. Using more appropriate dyes that are not contaminated by amlodipine fluorescence, namely Fluo-4, Cal 520 and Fura Red, we have consistently failed to detect Ca^2+^ influx at 0.5 µM or 20 µM amlodipine. Johnson et al. (8) reported a very small cytosolic Ca^2+^ rise to 0.5 µM amlodipine in Fluo-4 loaded cells. We do not have an explanation why our results differ from this earlier observation, but we note that numerous earlier studies all failed to observe Ca^2+^ influx in non-excitable cells to a range of LCCBs (22,26,37–43). It is important for future studies with amlodipine to avoid the use of Fura-2 and instead select a dye with longer excitation wavelengths such as Fluo-4, Cal 520 or Fura Red.

While we have been unable to measure any amlodipine-induced Ca^2+^ signals up to 20 µM, above this threshold we do detect effects on both intracellular Ca^2+^ release and calcium entry. Strikingly, these responses to amlodipine are concomitant with a dose-dependent accumulation of amlodipine fluorescence within cells, suggesting that intracellular accumulation might be the trigger for Ca^2+^ release. The effects of amlodipine on intracellular Ca^2+^ release fully deplete the thapsigargin-sensitive ER Ca^2+^ pool. This should lead to prominent store-operated Ca^2+^ entry. However, over the same concentration range, amlodipine inhibits CRAC channels, therefore preventing Ca^2+^ entry (16).

Amlodipine is largely charged at physiological pH. Seminal studies investigating the pH dependence of amlodipine block of L-type Ca^2+^ channels found that the drug–channel interaction was such that amlodipine was directly accessible to extracellular H^+^ (44). Subsequent work demonstrated that amlodipine blocked the channels but only when applied extracellularly. Intracellular perfusion of amlodipine was ineffective (45). Consistent with these findings, Hughes and Wijetunge (19) noted that amlodipine inserted into the outer leaflet of the plasma membrane at concentrations found therapeutically but, because of the polar side chain, was unable to diffuse across the plasma membrane. This would prevent any access to intracellular compartments at concentrations seen in patients. Exposure to higher concentrations, orders of magnitude greater than therapeutic levels, does result in some intracellular accumulation, but this is of questionable therapeutic relevance.

These earlier studies (19,44,45) are therefore inconsistent with the suggestion that amlodipine accumulates intracellularly at therapeutic concentrations to levels of tens of micromolar that are required to activate store operated Ca^2+^ entry (8,9). Such extensive accumulation within the ER is required for amlodipine to interact with STIM proteins and increase STIM dimer formation, as such interaction was seen with 20 µM amlodipine (8). Our new findings are relevant to this discussion. First, our data from cells isolated from chronic anti-hypertensive patients exposed to amlodipine for >15 years show intracellular accumulation is undetectable, and certainly much less than 1 µM. Our findings therefore do not support the view that amlodipine accumulates in the ER to the high µM levels required to elicit STIM dimerization. Second, accumulation to micromolar levels is only seen when extracellular concentrations of amlodipine are greater than 1 µM. Such high levels of intracellular accumulation would be highly problematic. At such concentrations, CCBs block multiple proteins including myosin light-chain kinase (46), phosphodiesterases (47) and affect Ca^2+^ATPase activity (47). Furthermore, numerous cytochrome P450 enzymes, which are responsible for metabolism of >75% of drugs in humans (48), are inhibited by dihydropyridines in the low micromolar range (49). A block of these enzymes should lead to profound drug–drug interactions in patients taking amlodipine, which is contrary to clinical experience. Because cytochrome P450 are integral membrane proteins mainly localized primarily in the ER (50), these findings support the view that amlodipine does not accumulate at the ER to the µM levels required to impair enzyme activity.

A further experimental issue that should be borne in mind in future studies is the problem of adding amlodipine and presumably other LCCBs to cell culture medium to study effects on cell proliferation and migration. We have found that amlodipine is heavily buffered by FBS, reducing the free concentration by probably 100-fold and at least >4-fold, if amlodipine binding deviates from expected linearity. This has important consequences. 0.5 µM amlodipine was the lowest concentration that evoked a cytosolic Ca^2+^ signal (8), although the response was miniscule. Store-operated Ca^2+^ entry evoked by 0.5 µM amlodipine in FBS was reported to increase cell proliferation through a mechanism that synergized with PDGF (8). However, the free amlodipine concentration in the culture medium is well below 0.5 µM, which was the threshold for activating CRAC channels. Therefore, the action of amlodipine in FBS together with PDGF is independent of store-operated Ca^2+^ entry.

Previously, we published a meta-analysis and prospective real-world study (16) that was consistent with numerous large-scale studies indicating that LCCBs are not associated with an increased risk of heart failure and that amlodipine is safe and effective (10–15). Our data here demonstrate that amlodipine affects calcium signals in multiple ways and in a wide variety of cells, but only at pharmacological concentrations. At concentrations relevant to therapeutic levels in patients, our data do not support the view that amlodipine activates the store-operated Ca^2+^ signaling pathway.

## Data Availability

Data and materials are available upon request to the corresponding author.
